# Effects of Linseed Supplementation on Milk Production, Composition, Odd- and Branched-Chain Fatty Acids, and on Serum Biochemistry in Cilentana Grazing Goats

**DOI:** 10.3390/ani12060783

**Published:** 2022-03-20

**Authors:** Nadia Musco, Raffaella Tudisco, Giulia Esposito, Piera Iommelli, Pajaree Totakul, Biagio D’Aniello, Pietro Lombardi, Ruggero Amato, Metha Wanapat, Federico Infascelli

**Affiliations:** 1 Department of Veterinary Medicine and Animal Production, University of Naples Federico II, 80137 Napoli, Italy; nadia.musco@unina.it (N.M.); tudisco@unina.it (R.T.); pietro.lombardi@unina.it (P.L.); ru.amato@outlook.com (R.A.); infascel@unina.it (F.I.); 2 Department of Veterinary Science, University of Parma, 43126 Parma, Italy; giulia.esposito@unipr.it; 3 Tropical Feed Resources Research and Development Center, Department of Animal Science, Faculty of Agriculture, Khon Kaen University, Khon Kaen 40002, Thailand; pajaree_totakul@hotmail.com (P.T.); metha@kku.ac.th (M.W.); 4 Department of Biology, University of Naples Federico II, 80137 Napoli, Italy; biagio.daniello@unina.it

**Keywords:** OBCFA, linseed, fat supplementation, milk, blood profile

## Abstract

**Simple Summary:**

Linseed has been utilized in ruminant nutrition to improve the production and quality of milk. In regard to the increasing concerns of consumers for the healthy aspects of foods as well as for animal welfare, in this research, we showed that linseed supplementation increased fat content but did not improve the milk concentration of odd- and branched-chain fatty acids (OBCFA), which are known to produce healthy effects in humans. Importantly, linseed did not show negative effects on animals’ health, suggesting it can be safely used in goats’ diet.

**Abstract:**

The purpose of this study was to investigate the effects of linseed supplementation on milk yield and quality, serum biochemistry and, in particular, to evaluate its possible effects on the production of odd- and branched-chain fatty acids (OBCFA) in the milk of Cilentana grazing goats. Twelve pregnant Cilentana dairy goats were divided into two groups (CTR, control, and LIN, linseed supplementation group). After kidding, the goats had free access to the pasture and both groups received a supplement of 400 g/head of concentrate, but the one administered to the LIN group was characterized by the addition of linseed (in a ratio of 20% as fed) to the ingredients. During the trial, milk samples were taken from April to August in order to evaluate milk production, composition, and fatty acid profile. In addition, blood samples were taken for evaluating the effects of linseed supplementation on goats’ health status. The health status of the goats was not influenced by the linseed supplementation, as confirmed by blood analyses. Concerning the effects on milk, the supplementation positively affected (*p* < 0.001) milk production and fat percentage and the fatty acid profile was markedly influenced by the lipid supplementation. In particular, milk from the LIN group was characterized by significantly lower concentrations of saturated fatty acids (FA; *p* < 0.001) and higher proportions of monounsaturated FA, polyunsaturated FA, and conjugated linoleic acids (CLAs) than milk from the CTR group (*p* < 0.001). In contrast, the OBCFA were negatively influenced by the linseed supplementation (*p* < 0.0001). Further studies are needed to test the effects of different fat sources and other nutrients on the diets.

## 1. Introduction

As reported by the FAO [1], the total world production of goat milk in 2017 reached 18.7 million tons and its consumption is considerable in some Mediterranean countries. In recent years, the interest in goat milk has increased because of its functional and dietary properties and high nutritional value [2,3,4]. In addition to the widely known compounds that can produce beneficial effects on consumer health, such as polyunsaturated fatty acids (PUFA), Omega−3, and conjugated linoleic acids (CLAs), milk from ruminants contain some minor but important components of fat, such as the odd- and branched-chain fatty acids (OBCFA). Intestinal absorption of microbially produced OBCFA and their absorption by the mammary gland led to the appearance of OBCFA in the milk fat of lactating dairy cows. Different groups of rumen microbial species have unique OBCFA profiles [5]. Cellulolytic bacteria mainly synthesize iso fatty acids (FA), while amylolytic bacteria produce high levels of anteiso and odd-chain linear FA and relatively low levels of iso FA [6]. Given that lipid supplementation can influence the various steps that determine the FA profile of milk, few data are available about how the OBCFA profile of milk may be influenced by the presence of additional lipids in the diet. Therefore, the milk OBCFA profile could potentially be used as a noninvasive method to predict some aspects of ruminal function.

Several studies aimed to discuss the linkage between animal diet and rumen microbiota, and the related effects on milk quality. The content of OBCFA in goat milk fat depends on the characteristics of the diet, such as the forage-concentrate ratio [7], the integration of vegetable oil [8], its interaction with forage level [9], and the percentage of physically effective neutral detergent fiber (NDF) [10]. In Italy, dairy goat farming systems are characterized by a high variability: from intensive indoor systems, mostly of specialized breeds, to semiextensive and extensive outdoor systems, with local breeds, depending on the economic importance of the supply chain and environmental and race specificities [11,12]. 

The integration of fat in the diet of lactating dairy cows is an approach used to improve the energy of the ration. In early-lactating cows, this feeding practice limits the amount of negative energy balance needed to support milk production [13]. It is well-documented that the inclusion of supplemental lipids in the diet can alter the rumen environment, thus influencing microbial fermentation and volatile fatty acid (VFA) production [14]. One of the most interesting lipid supplements is linseed (*Linum usitatissimum* L.) since it contains high levels of alfa-linoleic acid (ALA; 50% to 60% of total fatty acids, FA) and a lower concentration of linoleic acid (LA) and saturated fatty acids (SFA) compared to soybean, sunflowers, cottonseed, and corn [15]. Despite linseed supplementation being shown to affect milk production and composition, no studies have focused on its effect on the production of OBCFA. For this reason, the purpose of this study was to investigate a possible increasing effect of linseed on the production of odd- and branched-chain fatty acids in milk. Additionally, since energy changes and lipid supplementation may affect metabolism, mainly liver function, a serum biochemistry profile including liver and kidney markers was performed to assess possible adverse effects of linseed supplementation in Cilentana grazing goats. 

## 2. Materials and Methods

### 2.1. Animals, Diets, and Management

Twelve pregnant dairy goats (breed: Cilentana; calving: 3rd; body weight: 46.3 ± 1.2 kg) reared in a farm located in Salerno province (Italy), were equally divided into Control group (CTR) and linseed supplementation group (LIN), homogeneous for calving and milk production at the previous lactation (1260 ± 126 g/head/day). The experimental procedures were carried out at the Department of Veterinary Medicine and Animal Production (University of Napoli Federico II) and were approved by the local Bioethics Committee (protocol number: PG/2019/0070006). Goats were fed oat hay ad libitum, and concentrate in a ratio of 100, 200, and 300 g/head/day at 45, 30, and 15 days before kidding, respectively, using ultrasonography data to assess the state of pregnancy. After kidding (first week of March), every day, from 8:00 AM to 15.00 PM, the goats had free access to the pasture composed of 60% of Leguminosae and 40% of Graminae. In addition, both groups received 400 g/head/day of concentrate and the concentrate administered to the LIN group was characterized by the inclusion of linseed (20% a. f. within the ingredients). In order to keep the two diets isoproteic, a different amount and different ingredients were used for the control group (Table 1). During the experimental period, the goats from each group grazed in the daytime and were kept together in a barn after sunset to respect the habits of the farm and to avoid affecting animals’ feeding behavior. Each barn was equipped with individual self-closing feeders. 

Pasture samples were collected from four different areas (2.5 m^2^/area) and were cut at 3 cm from the ground. Then, the pasture samples were weighed and the four different samples from the different areas were dried using an air oven set at 65 °C, then were milled and stored until the analyses. According to Van Soest et al. [16] and AOAC [17], the chemical composition of feeds was analyzed, while the net energy was calculated as suggested by INRA [18]. Finally, according to Castro et al. [19], concentrates and pasture samples were analyzed using gas chromatography to determine the fatty acids profile (Table 1).

### 2.2. Milk Collection and Analysis

After kidding (first week of March), as in farm practice, milk was only suckled by kids, and by the end of April (final week), they completed weaning and goats were milked twice a day. Milk yield was recorded daily using a graduated cylinder after milking. Additionally, representative milk samples were collected monthly (for a total of 5 samples, from April to August) and stored at −20 °C until analysis. Then, the milk samples were analyzed for protein, fat, and lactose concentrations by the infrared method (Milkoscan 133B, Foss Matic, Hillerod, Denmark) standardized for goat milk. In addition, milk was extracted, methylated, and analyzed by liquid chromatography LCG as described by Tudisco et al. [20]. Briefly, milk fat was separated using a mixture of hexane and isopropane (3/2 *v*/*v*) [21]. The fatty acids methyl esters (FAMEs) were prepared by direct transesterification of the lipids with sulfuric acid and methanol (1:9, *v*/*v*) [22]. FA transmethylation and quantification were performed in the same way; additional standards for conjugated linoleic acid (CLA) isomers were purchased from Larodan (Larodan Fine Chemicals, AB, Limhamnsgardens Malmo, Sweden). The FAMEs were analyzed in a gas chromatograph (Agilent technologies, model 5890) fitted with an SP-2560 fused silica capillary column (100 m × 0.25 mm i.d. × 0.2 µm film thickness, Supelco, Inc., Bellefonte, PA, USA). The carrier gas, helium, was set at a constant pressure of 180 kPa, splitting flow of 50 mL/min, and injection volume of 1 µL. Column parameters: the initial temperature of the column was maintained at 170 °C for 15 min; then, with an increase of 5 °C/min, it was brought up to 240 °C. The total execution time was 64 min.

### 2.3. Blood Analysis

Five milliliters of blood were drawn at 7.00 am every 60 days (April, June, and August) from each goat via jugular venipuncture after a 12 h fast, and was collected into BD Vacutainer plastic tubes with clot activator and gel. Serum was obtained by centrifugation (1500× *g* for 15 min) and stored at −20 °C in 1.5 mL Eppendorf Safe-Lock Tubes until analysis. Serum samples were analyzed for the following: alanine amino transferase (ALT), aspartate amino transferase (AST), gamma glutamyl transferase (GGT), alkaline phosphatase (ALP), glucose (GLU), total protein (TP), creatinine (CREA), blood urea nitrogen (BUN), cholesterol (CHOL), and triglycerides (TRI). Analysis was performed using an automatic biochemical analyzer (AMS Auto lab, Diamond Diagnostics, West Point, UT, USA) using reagents from Spinreact (Girona, Spain).

### 2.4. Statistical Analysis

Feed data were analyzed using the one-way ANOVA with JMP software (version 11, PROC GLM, SAS 2000) according to the following model:

yij = µ + Sj + εij(1)

where y = single datum, µ = general mean, S = sampling effect (j = 5; April, May, June, July, August), and ε = residual error. 

Milk and blood data were analyzed using the two-way ANOVA with JMP software (version 11, PROC GLM, SAS 2000) according to the following model:

yijk = µ + Di + Sj + (DS)ij + εijk(2)

where y = single datum, µ = general mean, D = effect of the dietary treatment (i = 2; CTR and LIN), S = sampling effect (j milk = 5; April, May, June, July, August; j blood = 3; April, June, August), DS = interaction between dietary treatment × sampling effect, and ε = residual error. The mean was statistically compared using Tukey’s test. Differences were considered statistically significant at *p* < 0.05.

## 3. Results

**Figure 1:**
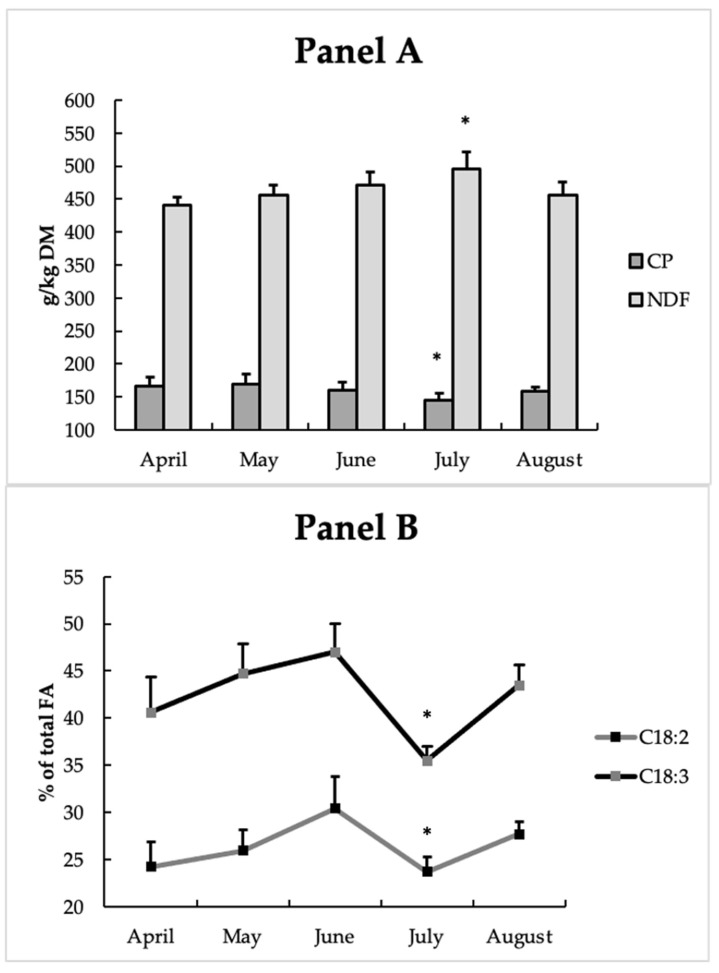
Pasture crude protein (CP) and neutral detergent fibre (NDF; **Panel A**) and fatty acid profile (**Panel B**) during the experimental period (mean ± SD). FA: fatty acids; DM: dry matter. Pasture composition: Leguminosae (in a ratio of 60% *Trifolium alexandrinum*, *Vicia* spp.) and Graminae (in a ratio of 40% *Bromus catharticus*, *Festuca arundinacea*, *Lolium perenne*). * *p* < 0.05.

As Figure 1 shows, the pasture’s chemical composition was affected by the month of sampling; in particular, NDF was significantly (*p* < 0.05) higher in July, and an opposite trend was observed for CP (*p* < 0.05). In addition, C18:2 and C18:3 were significantly (*p* < 0.05) lower in July compared to the other months of sampling.

**Figure 2:**
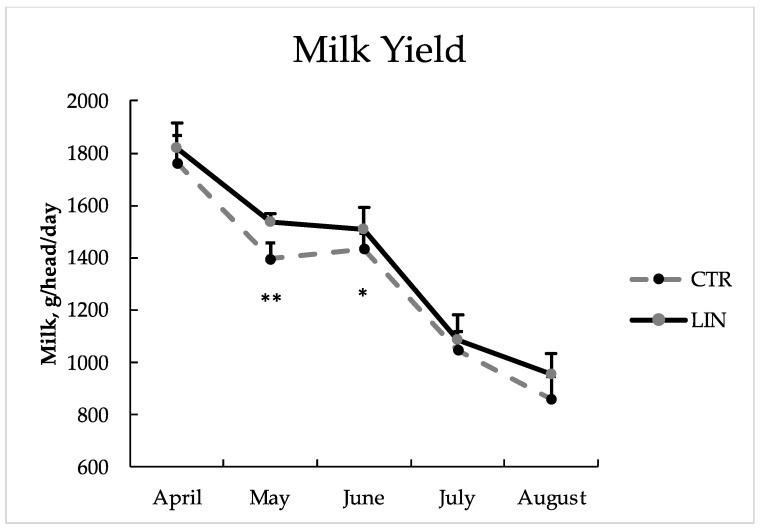
Goats’ milk production (mean ± SD) during the experimental period of control (CTR) and linseed supplementation (LIN) groups. ** *p* < 0.01; * *p* < 0.05.

The LIN group showed a significantly (*p* < 0.001) higher milk yield (Table 2), expressed as g/head/day, compared to the CTR group (1358 vs. 1291 for LIN and CTR, respectively) and, as Figure 2 shows, such a difference was particularly noticeable in May and June. Additionally, the control group produced significantly less milk fat compared to the treated group (*p* < 0.001; 3.05% vs. 4.10%). No differences between groups were observed for protein and lactose content. The significant (*p* < 0.001) differences registered for lactose content during the trial (time effect) reflect a physiological trend not ascribable to the different dietary treatment between groups.

**Figure 3:**
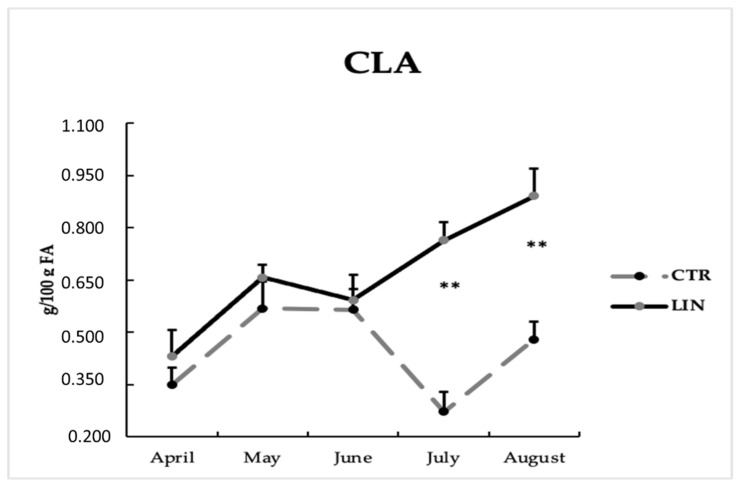
Goats’ CLAs during the experimental period (mean ± SD) of control (CTR) and linseed supplementation (LIN) groups. FA: fatty acids; CLA: conjugated linoleic acids. ** *p* < 0.01.

In Table 3, the milk fatty acid profile is reported. With the exception of the n6-n3 ratio and C18:2 (t10, c12) n6 content, all classes of fatty acids were significantly (*p* < 0.001) different between groups. The LIN group revealed a higher concentration of all classes of fatty acids, except for SFA, which was significantly (*p* < 0.001) higher in the CTR group (73.19 vs. 69. 47 g/100 g of total FA for CTR and LIN groups, respectively). CLAs were significantly (*p* < 0.001) higher in the LIN group compared to the CTR group (0.735 vs. 0.429 g/100 g of total FA), as Figure 3 shows.

Odd- and branched-chain fatty acid content is reported in Table 4. The total OBCFA was significantly affected by dietary treatment (*p* < 0.001; 3.21 vs. 2.84 g/100 g of total FA for CTR and LIN group, respectively) and an opposite trend was seen for iso C13:0 (0.028 vs. 0.034 for CTR and LIN group, respectively, *p* < 0.001).

**Figure 4:**
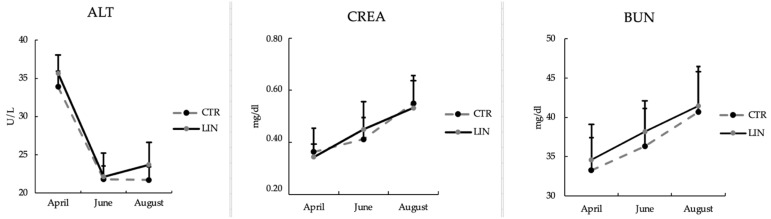
Indicators of liver and kidney status (mean ± SD) of goats during the experimental period of control (CTR) and linseed supplementation (LIN) groups. ALT: alanine amino transferase; CREA: creatinine; BUN: blood urea nitrogen.

The blood parameters are depicted in Table 5 and Figure 4. The fat supplementation did not determine significant changes in blood parameters’ levels, with the exception of triglycerides, which were significantly (*p* < 0.01) higher in the treated group compared to the control group (24.34 vs. 18.49 mg/dl). Despite no differences being observed between groups for the other parameters, liver and renal function seemed to be significantly (*p* < 0.05) affected by time; in fact, ALT significantly (*p* < 0.0001) decreased during the trial, whereas CREA and BUN significantly (*p* < 0.001) increased during the experimental period (Figure 4).

## 4. Discussion

As already evidenced in previous studies, linseed supplementation significantly improved milk yield in goats [23,24], as well as in dairy cows [25,26] and in dairy sheep [27]. On the contrary, other authors registered a similar milk yield with and without linseed supplementation in the diets [28,29,30]. In the present trial, a significantly higher fat production was registered in the experimental group, similar to Renna et al. [31], and in contrast with Loor et al. [32] and Almeida et al. [33], who found no differences in milk fat content with linseed supplementation in the diets. As reported by Abuelfatah et al. [34], the inclusion of linseed in the diet of goats appears to be able to increase the molar proportion of acetate in the rumen, which may be responsible for the fat increase in milk. Indeed, these differences could be attributable to the shape of the administered linseed. As reported by Chilliard et al. [35], the treatment to which linseed is often subjected (i.e., micronization, extrusion, or heat treatments) can affect milk production and milk fat concentration, sometimes even leading to a decrease. In fact, these treatments affect the oil release rate compared to the whole seeds, thus leading to an increase in the production of trans FA in the rumen and, therefore, a decrease in the fat content of the milk [36]. 

Milk protein and lactose were unaffected by linseed supplementation, as previously reported by Chilliard et al. [35]. As reported by Sarrazin et al. [37], the levels of fat inclusion in the diet can affect milk production; moderate inclusion levels increase milk production by improving feed efficiency while higher inclusion levels negatively affect feed intake, consequently depressing ruminal function and decreasing milk yield [38]. As reported by Chilliard et al. [39], when lipids are ingested or directly infused into the rumen, the decrease in feed intake is even greater. These negative effects are proportional to the polyunsaturated fatty acid content of the lipids. These effects on feed intake have long since been attributed to the negative effects of lipid infusion on rumen digestion [40] or to palatability problems associated with high-fat diets.

The milk fatty acid profile was markedly influenced by lipid supplementation. In particular, milk from the LIN group was characterized by lower concentrations of SFA and higher proportions of MUFA, PUFA, and CLAs than milk from the CTR group, as previously observed by Nudda et al. [23] and Tudisco et al. [24]. 

The milk content of CLAs increased in the experimental group. As reported by Dilzer and Park [41], in the last twenty years, CLAs have acquired the interest of the scientific community for their anticarcinogenic effects, but they are also known to reduce the risk of cardiovascular diseases, reduce body fat, and modulate immune and inflammatory responses. CLAs are intermediates of the biohydrogenation in the rumen mammary [42] of C18:2 cis-9 cis-12 and C18: 3 linolenic acids, which is present in high concentrations in linseed. In addition, CLAs were also a product of a Δ9-desaturase, the stearoyl-CoA desaturase (SCD) in the mammary gland [43]. Other studies reported an increase in CLAs in milk in different ruminants’ species, such as Bernard et al. [28] in goats, Bu et al. [44] and Ferlay et al. [45] in cows, and Gomez-Cortes et al. [46] and Kholif et al. [47] in ewes. Chillard et al. [29] reported that CLAs’ concentration in goat milk after the administration of linseed and sunflower oil achieved a peak after two weeks of supplementation and persisted for at least ten weeks before decreasing. Contrastively, in dairy cows, Roy et al. [48] reported that the supplementation with linseed oil led to an increase in CLAs in milk that persisted throughout lactation. In our trial, we recorded a similar trend for the first three months of observation (April, May, and June) and a significant increase in CLAs in the treated group (Figure 3) during the deterioration of the pasture (July), particularly regarding the two precursors of CLAs (C18:2 and C18:3). Thus, we may speculate that, in grazing goats, linseed supplementation produces a beneficial effect when the pasture has a low PUFA concentration.

This study examined the relationship between milk OBCFA and dietary composition to improve our knowledge of the interactions occurring with lipid supplementation. The dietary treatment affected only a few OBC fatty acids and, except for C13:0 iso, linseed reduced the OBCFA concentration in milk. These results could be attributable to the negative effects of the fat supplement on the rumen bacteria. The inhibitory effects of long-chain fatty acids on rumen bacteria are well-documented and are greater by increasing the unsaturation of long-chain fatty acids. Importantly, there are several differences between bacteria, for example, the growth of cellulolytic strains is lower than that of amylolytic ones [49].

Hence, when the fat source contains high amounts of C18: 2 n-6 and C18: 3 n-3 (e.g., sunflower oil and linseeds, respectively), effects on a bacterial population can be expected. As reported by Maia et al. [50] and Cívico et al. [51], linseed oil produces an inhibitory effect on some rumen bacterial populations and, particularly, cellulolytic bacteria are negatively affected by PUFA. Indeed, the main bacteria susceptible to unsaturated FA are the cellulolytic ones, particularly to alfa-linolenic acid [52], which is the main fatty acid supplied by linseed oil. Additionally, cellulolytic bacteria participate in the predominant rumen biohydrogenation pathway; hence, it might be expected that linseed oil could positively affect the rumen biohydrogenation intermediate levels in milk fat while lowering its iso FA content according to the analysis of milk OBCFA. Dairy cows that were fed supplemental fat rich in C18:3 n-3 [53,54] had lower proportions of milk OBCFA. In agreement with this hypothesis, and according to our results, Bernard et al. [28] found a decrease in the majority of iso FA content in milk fat relating to an increase in the proportions of total trans C18: 1 and total conjugate C18: 2 by adding linseed oil to the diet of dairy goats. The nutritional treatment significantly reduced the milk concentrations of C15: 0 and C17: 0 and this could be considered a nonpositive result. In fact, these fatty acids are considered biomarkers of rumen microbial fermentation and microbial de novo lipogenesis and the mammary gland plays a pivotal role in their synthesis [55]. Recently, the importance of C15: 0 and C17: 0, in terms of human health, has been highlighted. These fatty acids are able to reduce the risk of cardiovascular diseases [56] and the incidence of diabetes type 2 [57]; in addition, C15:0 reduces the inflammatory state [58]. Humans are not able to synthetize these fatty acids, but they can intake it with dairy products.

During the experimental period, the pasture underwent a progressive modification due to the phenological stage of the plants, which led to changes in its chemical characteristics. As lactation progressed, there was a deterioration of the nutritional quality of the pasture, as can be seen in Figure 1, showing an increase in fiber and a reduction in the content of proteins and fatty acids, the concentration of which decreased in mature grass. Along with the lactation progress, there was an increase in neutral detergent fiber (NDF) in the pasture, thus suggesting a positive association between OBCFA and dietary fiber, as previously observed by Bas et al. [59] in dairy goats. These researchers highlighted dietary NDF as the most important factor of variation in the lipid composition of bacteria.

Concerning the effects of the linseed supplementation on goats‘ health during the experimental period, some serum biomarkers of renal and hepatic function changed. In particular, ALT decreased, while creatinine and bilirubin increased over time. We can observe that the temporal decrease in ALT shown in both groups followed the same trend of the lactation curve, as previously reported by Nudda et al. [23] in goats. The temporal increases in creatinine and bilirubin during the study were also observed in both groups. Generally, an increase in creatinine is associated with a decrease in renal function but, in this case, the registered values were not outside the physiological range reported for this species [60]. This consideration could indicate that no kidney lesions occurred, and since the creatinine concentration in the blood is generated by muscle metabolism, its increase during lactation could be due to a normal muscle turnover.

The differences found between groups for triglycerides are difficult to explain and, although the LIN group had a higher triglycerides value than the CTR group, both results reflect a physiological rather than a pathological condition, and are in line with previous studies conducted under the same experimental conditions [61].

## 5. Conclusions

Linseed supplementation was able to improve milk yield and fat content but not the milk concentration of the odd- and branched-chain fatty acids. Thus, the trial hypothesis has been only partially confirmed. Importantly, serum biochemistry showed no negative effects of linseed supplementation at the tested intake. Since the fatty acid composition in milk, as well as in other dairy products, has nutritional benefits in terms of human health, further studies using different experimental conditions might reveal a possible milk OBCFA improvement by linseed supplementation in goats’ diet.

## Figures and Tables

**Table 1:** Chemical composition and fatty acid profile (mean ± SD) of feeds administered to goats.

Chemical Composition g/kg DM	CTR Concentrate	LIN Concentrate	Linseed	Oat Hay	Pasture
CP	177.4 ± 4.75	178.6 ± 3.69	242.3 ± 5.83	88.4 ± 5.08	160 ± 20.04
EE	28.70 ± 2.66	67.40 ± 1.36	350.2 ± 7.45	19.4 ± 1.84	20.3 ± 1.40
NDF	262.5 ± 6.13	266.9 ± 8.51	259.3 ± 7.12	594.5 ± 12.35	465.9 ± 50.12
ADF	98.60 ± 6.90	101.4 ± 7.04	112.1 ± 3.41	312.7 ± 9.270	338.4 ± 15.25
ADL	26.70 ± 1.33	27.30 ± 1.51	54.10 ± 2.11	39.6 ± 2.98	50.0 ± 7.19
PDIN g/kg DM	116.0 ± 3.66	100.5 ± 2.90	168.3 ± 3.86	62.3 ± 4.52	112.2 ± 12.31
PDIE g/kg DM	100.8 ± 2.72	104.4 ± 3.11	133.6 ± 2.98	64.5 ± 3.47	76.4 ± 9.90
Fatty acid profile % of total FA					
SFA	28.4 ± 1.13	26.6 ± 1.22	10.9 ± 0.31	.	17.7 ± 2.22
MUFA	15.8 ± 0.65	14.3 ± 0.25	22.2 ± 1.64	.	6.3 ± 0.74
PUFA	55.8 ± 2.61	59.12 ± 4.16	66.9 ± 2.51	.	76.0± 8.36
C18:2	48.7 ± 2.34	37.9 ± 2.19	13.7 ± 0.78	.	26.4 ± 6.70
C18:3	3.50 ± 0.12	18.3 ± 1.43	53.2 ± 4.82	.	41.9 ± 5.76
**Concentrates ingredients** **% as fed**	**CTR**	**LIN**			
	Soft wheat bran 26.6; Corn meal 15.0; Sunflower meal 14.5; Dried pulp beet 12.0; Fava bean 10.6; Corn gluten feed 7.0; Dried citrus pulp 6.5; Molasses 5.6; Vitamin-mineral premix 2.2	Soft wheat bran 30.0; Corn meal 23.0; Linseed 20.0; Dried citrus pulp 10.0; Dried pulp beet 8.0; Corn gluten feed 7.0; Vitamin–mineral premix 2.0.			

CTR: control group; LIN: linseed supplementation group; CP: crude protein; EE: ether extract; ADF: acid detergent fiber; ADL: acid detergent lignin; PDIN: protein digested in the small intestine when rumen-fermentable nitrogen is limiting; PDIE: protein digested in the small intestine when rumen-fermentable energy is limiting; MUFA: monounsaturated fatty acids; NDF: neutral detergent fiber; PUFA: polyunsaturated fatty acids; SFA: saturated fatty acids; UFL: unit feed for lactation.

**Table 2:** Goats’ milk production and composition (mean value, g/head/day) of control (CTR) and linseed supplementation (LIN) groups.

	Milk Production		Fat		Protein		Lactose	
	**CTR**	**LIN**	**CTR**	**LIN**	**CTR**	**LIN**	**CTR**	**LIN**
Mean value	1291.38	1358.72	40.77	57.13	42.28	46.75	52.96	56.24
April	1763.33	1818.33	56.42	63.28	58.90	59.46	75.47	84.01
May	1395.20	1536.39	51.34	72.52	45.76	52.85	61.39	65.60
June	1434.17	1506.67	45.75	70.51	44.89	50.32	56.22	60.27
July	1046.67	1085.21	30.25	37.77	33.28	35.70	40.30	41.13
August	858.33	953.33	23.43	41.37	28.67	34.32	33.73	35.27
RMSE	86.004		8.14		3.74		2.81	
***p*-value**								
Group	***		***		NS		NS	
Time	***		***		NS		***	
GxT	NS		NS		NS		*	

*** *p* < 0.001; * *p* < 0.05. NS: not significant; RMSE: root mean square error.

**Table 3:** Goats’ milk fatty acid profile (mean value, g/100 g of total fatty acids) of control (CTR) and linseed supplementation (LIN) groups.

	CTR	LIN	RMSE	*p*-Value		
				Group (G)	Time (T)	GxT
SFA	73.19	69.47	3.452	***	***	NS
MUFA	23.43	26.10	2.994	***	***	NS
PUFA	3.32	4.20	0.454	***	***	***
PUFA n3	1.16	1.38	0.177	***	***	***
PUFA n6	1.81	2.20	0.263	***	***	***
n6/n3	1.54	1.63	0.239	NS	***	***
C18:2 (c9, t11)	0.338	0.610	0.1648	***	**	**
C18:2 (t10, c12) n6	0.091	0.108	0.1184	NS	NS	NS
CLAs	0.429	0.735	0.243	***	**	**

SFA: saturated fatty acids; MUFA: monounsaturated fatty acids; PUFA: polyunsaturated fatty acids; CLAs: conjugated linoleic acids. *** *p* < 0.001; ** *p* < 0.01; NS: not significant; RMSE: root mean square error.

**Table 4:** Goats’ odd- and branched-chain fatty acid content (mean value, g/100 g of total fatty acids) of control (CTR) and linseed supplementation (LIN) groups.

	CTR	LIN	RMSE	*p*-Value		
				Group (G)	Time (T)	GxT
iso C13:0	0.028	0.034	0.007	***	NS	NS
iso C14:0	0.088	0.094	0.013	NS	NS	NS
iso C15:0	0.176	0.193	0.038	NS	NS	NS
iso C16:0	0.024	0.029	0.037	NS	*	NS
iso C17:0	0.027	0.034	0.049	NS	**	NS
anteiso C:13	0.0032	0.0040	0.004	NS	NS	NS
anteiso C:15	0.363	0.383	0.006	NS	NS	NS
anteiso C:17	0.391	0.404	0.083	NS	NS	NS
C15:0	0.823	0.609	0.053	***	***	***
C17:0 + cis9 C17:1	0.924	0.676	0.091	***	***	***
Total OBCFA	3.21	2.84	0.203	***	***	***

OBCFA: odd- and branched-chain fatty acids. *** *p* < 0.001; ** *p* < 0.01; * *p* < 0.05; NS: not significant; RMSE: root mean square error.

**Table 5:** Goats’ serum biochemistry (mean value) of control (CTR) and linseed supplementation (LIN) groups.

	AST	ALT	GGT	ALP	GLU	TP	CREA	BUN	CHOL	TRY
	U/L	U/L	U/L	U/L	mg/dL	g/dL	mg/dL	mg/dL	mg/dL	mg/dL
CTR	64.74	20.94	34.55	208.17	51.57	6.97	0.500	36.74	61.14	18.49
LIN	68.28	22.07	36.44	215.63	54.12	6.95	0.422	38.07	61.52	24.34
RMSE	7.333	2.473	5.121	39.542	5.934	0.564	0.142	5.237	8.261	4.852
Group (G)	NS	NS	NS	NS	NS	NS	NS	NS	NS	**
Time (T)	NS	***	NS	NS	NS	NS	**	**	NS	NS
GxT	NS	NS	NS	NS	NS	NS	*	NS	NS	*

AST: aspartate amino transferase; ALT: alanine amino transferase; GGT: gamma glutamyl transferase; ALP: alkaline phosphatase; GLU: glucose; TP: total protein; CREA: creatinine; BUN: blood urea nitrogen; CHOL: cholesterol; TRY: triglycerides. *** *p* < 0.001; ** *p* < 0.01; * *p* < 0.05; NS: not significant; RMSE: root mean square error.

## Data Availability

The data presented in this study are available on request from the corresponding author.
